# Efficacy of oblique pulling manipulation combined with adjunct therapies (massage/acupuncture/herbal medicine/injection) for lumbar disc herniation: a systematic review and meta-analysis of pain and functional outcome

**DOI:** 10.3389/fneur.2025.1700862

**Published:** 2026-01-19

**Authors:** Yi Li, Wenhui Li, Yanting Zhang, Meiyu Zhu, Xiaorong Jiang, Xiuqun Cai

**Affiliations:** 1 Health Management Center, Jinjiang Municipal Hospital (Shanghai Sixth People’s Hospital Fujian), Jinjiang, China; 2 Department of Health Management Center, Xiang'an Hospital of Xiamen University, Xiamen, China; 3 Department of Outpatient Services, Jinjiang Municipal Hospital (Shanghai Sixth People’s Hospital Fujian), Jinjiang, China; 4 Health Management Center, Jinjiang Municipal Hospital Jinnan Branch, Jinjiang, China; 5 Department of Management, Jinjiang Municipal Hospital Jinnan Branch, Jinjiang, China

**Keywords:** acupuncture, Chinese herbal medicine, injection therapy, massage, oblique pulling manipulation, traditional Chinese medicine

## Abstract

**Background:**

This study aimed to systematically evaluate the clinical efficacy of oblique pulling manipulation and its combination with massage, acupuncture, Chinese herbal medicine, and injection therapy in lumbar disc herniation (LDH).

**Methods:**

The meta-analysis followed the Preferred Reporting Items for Systematic Reviews and Meta-Analyses (PRISMA) guidelines and was registered with PROSPERO (CRD420251107984). A comprehensive search was conducted in databases including China National Knowledge Infrastructure (CNKI), Wanfang Database, Chinese Scientific Journals Database (VIP), Chinese Biomedical Literature Database (CBM), PubMed, EMBASE, Web of Science, and the Cochrane Library until June 2025. All statistical analyses were conducted using Review Manager 5.4.1. Dichotomous outcomes were summarized as odds ratios (ORs) with 95% confidence intervals (CIs), and continuous outcomes were summarized as standardized mean differences (SMDs) with 95% CIs.

**Results:**

A total of 26 studies comprising 2,766 patients with lumbar disc herniation were included. The results of the meta-analysis revealed that oblique pulling manipulation and its combination with massage, acupuncture, Chinese herbal medicine, and injection therapy in LDH significantly improved the effective rate and cure rate in patients with LDH. In addition, oblique pulling manipulation significantly improved the Japanese Orthopedic Association (JOA) score, and oblique pulling manipulation combined with massage or acupuncture decreased the Oswestry Disability Index (ODI).

**Discussion:**

Oblique pulling manipulation and its combination with massage, acupuncture, Chinese herbal medicine, and injection therapy improved efficacy and cure rates in patients with lumbar disc herniation. Future research should focus on standardizing treatment protocols and extending follow-up periods to confirm long-term safety and effectiveness, thereby ensuring broader applicability and better patient outcomes.

**Systematic review registration:**

https://www.crd.york.ac.uk/prospero/CRD420251107984, identifier: CRD420251107984.

## 1 Introduction

Lumbar disc herniation (LDH) is a syndrome caused by the degeneration of intervertebral discs, leading to annular disruption and nucleus pulposus protrusion that irritates nerve roots, manifesting as low back pain and sciatica (1, 2). Clinical manifestations include radicular pain, dermatomal numbness, motor weakness, cauda equina syndrome, or non-radiating referred pain mimicking visceral pathologies (3). The global annual incidence of LDH is 5 per 1,000 adults (4), and approximately 70–85% of people experience low back pain during their lifetime, which substantially impairs their health, quality of life, and work capacity (5). It places significant financial and logistical strain on healthcare systems, driving up treatment expenditures and resource demands. Although clinical studies have shown the possibility of spontaneous absorption of LDH, the difference in efficacy between conservative and surgical treatment is still controversial (6). Approximately 60–90% of LDH patients experience symptom relief through conservative interventions alone (7, 8).

In traditional Chinese medicine (TCM), manual therapy is one of the most commonly used complementary therapies for LDH, and oblique pulling manipulation is a typical manual therapy (9). Multiple clinical series have demonstrated its clinical practicability and effectiveness, with a reported effective rate as high as 93% (10–18). Despite widespread use, the evidence for oblique pulling manipulation remains fragmented. In addition to oblique-pulling manipulation, integrated TCM treatment for LDH typically incorporates therapeutic massage, acupuncture, Chinese herbal medicine, and injection therapy to relieve lumbar and leg pain and reduce neuroinflammatory processes, creating a multimodal framework that improves both short-term analgesia and long-term functional recovery in LDH. However, it is difficult to reach a firm efficacy conclusion for the combination TCM treatment due to the limited quality and the small sample sizes of previous studies. In this systematic review, we aimed to evaluate the clinical efficacy of oblique pulling manipulation and its combination with massage, acupuncture, Chinese herbal medicine, and injection therapy in LDH and, specifically, to compare cure rates and JOA scores across combined therapies.

## 2 Methods

This study was conducted following the Preferred Reporting Items for Systematic Reviews and Meta-analyses (PRISMA) guidelines. The protocol has been registered on PROSPERO, under the number CRD420251107984.

### 2.1 Literature search strategy

A systematic literature search was conducted in eight databases, including the China National Knowledge Infrastructure (CNKI), Wanfang Database, Chinese Scientific Journals Database (VIP), Chinese Biomedical Literature Database (CBM), PubMed, EMBASE, Web of Science, and the Cochrane Library. The retrieval period encompassed the entire duration of the databases up to 30 June 2025. Randomized controlled trials involving patients with lumbar disc herniation (LDH) treated with oblique pulling manipulation alone or in combination with other therapies were identified according to PICOS criteria: participants (P) with LDH; intervention (I) with oblique pulling manipulation ± adjunct therapies; comparison (C) with non-oblique pulling interventions; outcomes (O) including cure rate, overall effective rate, Japanese Orthopaedic Association (JOA) score, visual analogue scale (VAS), and Oswestry Disability Index (ODI); and study design (S) restricted to randomized controlled trials. The following search terms were used: “oblique pulling OR oblique pulling manipulation OR rotatory manipulation OR spinal manipulation” AND “LDH OR LDH OR lumbar radiculopathy OR sciatica OR intervertebral disc displacement” AND “traditional Chinese medicine OR TCM OR massage OR acupuncture OR herbal medicine OR injection therapy.”

### 2.2 Inclusion and exclusion criteria

The following inclusion criteria were applied: studies must have been randomized controlled trials (RCTs) enrolling adult patients (≥18 years) with a clear clinical and/or radiological diagnosis of LDH, comparing oblique pulling manipulation alone or combined with massage, acupuncture, Chinese herbal medicine, or injection therapy against non-oblique pulling interventions; studies must report clearly defined sample sizes, patient selection and diagnostic criteria, and intervention and control methods; count data (cure rate, overall effective rate) must be available or derivable to calculate odds ratios (ORs), and continuous outcomes (JOA score, VAS, and ODI) must be reported with means, standard deviations, and sample sizes; and studies must be published as full-text, peer-reviewed articles. The exclusion criteria comprised duplicate publications or overlapping data; flawed study design or low methodological quality; incomplete, unclear, or irretrievable outcome data; lack of count data and unavailable mean, standard deviation, or sample size for continuous outcomes; incorrect or uncorrectable statistical methods; non-RCT designs such as case reports, case series, reviews, expert opinions, animal studies, and meta-analyses; conference abstracts, theses, dissertations, expert lectures, or government reports; and studies enrolling patients with contraindications to oblique pulling manipulation.

### 2.3 Risk of bias assessment

Risk of bias was independently evaluated by two reviewers using the Cochrane Risk of Bias Tool for Randomized Trials (RoB 2.0). Assessment covered five domains: the randomization process, deviations from intended interventions, missing outcome data, outcome measurement, and selection of the reported result, as well as an overall bias judgment. Disagreements were resolved through discussion and, if necessary, by consulting a third researcher. Each study was then classified in each domain and assigned an overall judgement of low risk, some concerns, or a high risk of bias.

### 2.4 Data extraction

Two independent reviewers used a standardized data—extraction sheet to collect and cross-check the following trial data: patient demographics (mean age, sex distribution, total sample size, and disease duration), intervention details (specific oblique pulling manipulation protocols alone or combined with massage, acupuncture, Chinese herbal medicine, or injection therapy, and total treatment duration), and key outcome measures [cure rate, efficacy rate, Japanese Orthopaedic Association (JOA) score, visual analogue scale (VAS) score, and Oswestry Disability Index (ODI) score]. Any discrepancies were resolved through discussion or, if necessary, through third-party adjudication. The finalized data were then organized into a comprehensive summary table to facilitate heterogeneity assessment and the pooled effect-size analysis.

### 2.5 Statistical analysis

All statistical analyses were conducted using Review Manager 5.4.1. Dichotomous outcomes were summarized as odds ratios (ORs) with 95% confidence intervals (CIs), and continuous outcomes as standardized mean differences (SMDs) with 95% CIs. Inter-study heterogeneity was assessed by Cochran’s *Q* test (*p* < 0.05 indicating significant heterogeneity) and quantified using the *I*^2^ statistic, with *I*^2^ ≤ 50% deemed low heterogeneity and *I*^2^ > 50% indicating substantial heterogeneity. A fixed-effects model was applied when heterogeneity was low; otherwise, a random-effects model was used. Sensitivity analyses, including the leave-one-out approach and the exclusion of studies at high risk of bias, were performed to assess the robustness of the pooled estimates. Publication bias was examined through funnel plot inspection and Egger’s regression test, with a *p*-value of <0.05 denoting significant bias. All hypothesis tests were two-sided, and statistical significance was set at a *p*-value of <0.05.

## 3 Results

### 3.1 Screening strategy

**Figure 1:**
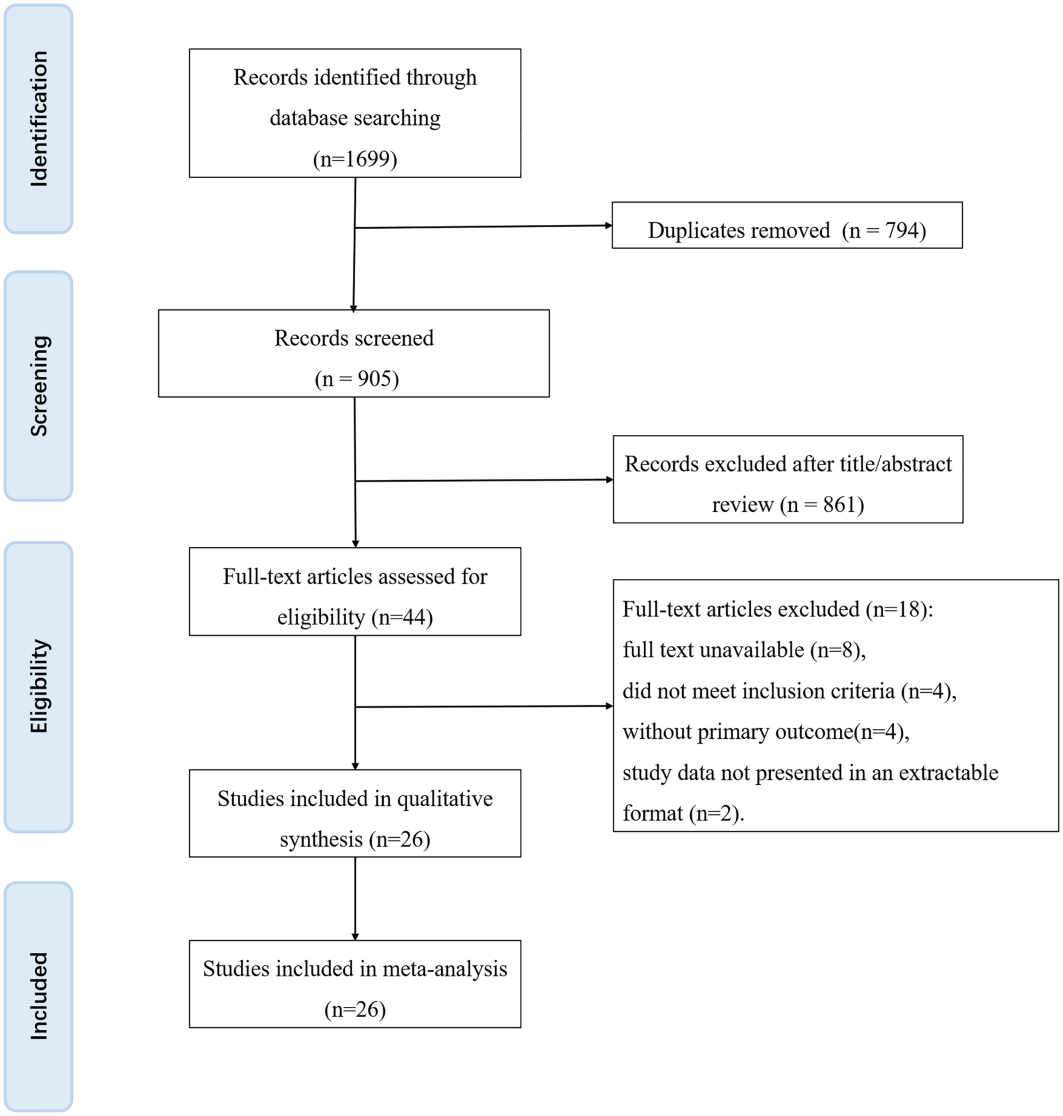
Flow diagram.

A total of 1,699 records were retrieved from the databases. After duplicate entries were removed, 2 researchers independently screened titles and abstracts to exclude irrelevant studies, yielding 44 articles for full-text assessment. A total of 18 studies were excluded for the following reasons: 8 were unavailable in full text, 4 failed to meet the predefined inclusion criteria, 4 did not report any primary outcomes, and 2 presented data in a non-extractable format. Consequently, 26 randomized controlled trials (RCTs) were included in the meta-analysis (Figure 1).

### 3.2 Characteristics of included studies

A total of 26 studies enrolled 2,766 patients with lumbar disc herniation (LDH), of whom 1,409 received oblique pulling manipulation interventions and 1,357 served as the control group. Interventions were classified into five types: oblique pulling manipulation, oblique pulling manipulation combined with massage, oblique pulling manipulation combined with acupuncture, oblique pulling manipulation combined with Chinese herbal medicine, and oblique pulling manipulation combined with injection therapy. The detailed characteristics of the included studies are summarized in Tables 1, 2.

**Table 1:** Characteristics of included studies.

Author	Age (y)		Sex (M/F)		Sample size		Disease duration		Intervention methods	Intervention time
	Treatment group	Control group	Treatment group	Control group	Treatment group	Control group	Treatment group	Control group		
Li and Sun (10)	38.57 ± 2.23	38.55 ± 2.33	25/26	24/25	51	49	0.4 ~ 5	1.8 ~ 6	④	<1 month
Wang (36)	57.36 ± 6.34	58.05 ± 6.68	25/15	24/16	40	40	0.04 ~ 6	0.04 ~ 7	①	1 month
Wang and Xing (21)	47.06 ± 8.51	52.10 ± 2.34	15/15	13/17	30	30	1.71 ± 0.40	1.07 ± 0.39	②	<1 month
Xiang et al. (26)	45.30 ± 9.23	44.61 ± 9.60	16/14	17/13	30	30	0.17 ~ 6	0.25 ~ 5	③	<1 month
Zhang et al. (22)	40.03 ± 10.40	40.48 ± 11.76	13/16	14/15	29	29	0.02 ~ 0.16	0.03 ~ 0.13	②	<1 month
Yang et al. (29)	42.13 ± 10.03	42.33 ± 8.99	13/17	14/16	30	30	N/A	N/A	④	<1 month
Feng et al. (11)	49.92 ± 6.69	49.28 ± 6.40	25/25	26/25	50	51	0.58 ~ 2	0.67 ~ 2	①	1 month
Feng (12)	55.86 ± 6.74	55.58 ± 6.69	22/26	23/25	48	48	0.51 ± 0.11	0.52 ± 0.11	①	<1 month
Huang (13)	39.21 ± 2.06	38.67 ± 1.13	56/34	50/40	90	90	N/A	N/A	①	<1 month
Zhong et al. (37)	52.10 ± 6.80	51.50 ± 7.60	17/13	16/14	30	30	0.25 ~ 2	0.17 ~ 4	③	<1 month
Zhuang (38)	56.54 ± 6.23	57.30 ± 7.30	28/15	30/13	43	43	2.85 ± 0.71	2.85 ± 0.67	②	<1 month
Hu (39)	44.80 ± 2.60	45.20 ± 3.10	34/18	37/15	52	52	N/A	N/A	③	<1 month
Xue (14)	42.08 ± 6.08	42.65 ± 6.82	32/18	30/20	50	50	N/A	N/A	①	1 month
Liu et al. (40)	34.47 ± 7.65	34.68 ± 7.46	27/23	28/22	50	50	0.07 ± 0.02	0.07 ± 0.02	③	<1 month
Wei et al. (41)	33.80 ± 3.70	34.20 ± 4.40	25/15	26/14	40	40	0.01 ~ 1.33	0.01 ~ 1.42	⑤	1 month
Huang and Jing (32)	43.30 ± 2.90	45.20 ± 3.40	21/22	19/24	43	43	0.17 ~ 3.08	0.25 ~ 3	⑤	1 month
Qu (42)	20 ~ 64		47/15	Sep-39	62	48	N/A	N/A	②	<1 month
Chen et al. (43)	21 ~ 65	23 ~ 65	22/18	24/16	40	40	0.01 ~ 1.92	0.02 ~ 3.17	②	<1 month
He et al. (44)	44	42	49/41	48/42	90	90	0.92	0.83	②	<1 month
Song and Luo (45)	22 ~ 62	23 ~ 61	27/13	26/14	40	40	0.17 ~ 3	0.17 ~ 2.5	③	<1 month
Zhang et al. (46)	25 ~ 54	27 ~ 53	14/18	15/16	32	31	0.5 ~ 10	1 ~ 10	②	<1 month
Zhou et al. (15)	43.60 ± 11.78	45.26 ± 11.60	21/19	25/17	40	42	N/A	N/A	①	<1 month
Ma and Zhang (16)	32	31.3	35/31	33/33	66	66	0.03 ~ 6	0.01 ~ 6.5	①	1 month
Yao et al. (30)	18 ~ 49		42/18	46/14	60	60	N/A	N/A	④	<1 month
Zhao (17)	42.3	43.5	55/30	35/28	85	63	0.08 ~ 6	0.08 ~ 5	①	<1 month
Lai (47)	20 ~ 75		N/A	N/A	188	172	0.08 ~ 12	N/A	⑤	<1 month

Y, year; M, male; F, female; ①, oblique pulling manipulation; ②, oblique pulling manipulation combined with massage; ③, oblique pulling manipulation combined with acupuncture; ④, oblique pulling manipulation combined with traditional Chinese medicine; ⑤, oblique pulling manipulation combined with injection therapy.

**Table 2:** Characteristics and efficacy endpoints of trials evaluating the TCM oblique-splinting method alone or in combination with four adjunct therapies for lumbar disc herniation.

Author	Cure rate				Effective rate				After treatment					
	Treatment group		Control group		Treatment group		Control group		JOA score		VAS score		ODI score	
	Cured	Uncured	Cured	Uncured	Effective	Ineffective	Effective	Ineffective	Treatment group	Control group	Treatment group	Control group	Treatment group	Control group
Li and Sun (10)	23.00%	28.00%	14.00%	35.00%	50.00%	1.00%	37.00%	12.00%	N/A	N/A	N/A	N/A	N/A	N/A
Wang (36)	11.25%	38.75%	12.50%	37.50%	40.00%	10.00%	42.50%	7.50%	13.29 ± 2.01	13.45 ± 1.70	3.82 ± 0.73	2.75 ± 0.64	33.58 ± 5.83	32.39 ± 5.29
Wang and Xing (21)	8.33%	41.67%	1.67%	48.33%	46.67%	3.33%	38.33%	11.67%	N/A	N/A	1.50 ± 1.01	2.43 ± 1.31	11.18 ± 3.43	16.43 ± 5.17
Xiang et al. (26)	30.00%	20.00%	21.67%	28.33%	46.67%	3.33%	36.67%	13.33%	N/A	N/A	N/A	N/A	8.06 ± 1.40	9.79 ± 1.52
Zhang et al. (22)	1.72%	48.28%	5.17%	44.83%	39.66%	10.34%	48.28%	1.72%	20.86 ± 4.44	23.45 ± 3.27	3.55 ± 2.10	2.24 ± 2.03	N/A	N/A
Yang et al. (29)	18.33%	31.67%	10.00%	40.00%	46.67%	3.33%	40.00%	10.00%	N/A	N/A	2.03 ± 0.56	2.93 ± 0.91	N/A	N/A
Feng et al. (11)	23.76%	25.74%	17.82%	32.67%	48.51%	0.99%	43.56%	6.93%	23.34 ± 2.18	19.26 ± 2.44	2.08 ± 0.53	3.86 ± 0.48	N/A	N/A
Feng (12)	N/A	N/A	N/A	N/A	N/A	N/A	N/A	N/A	25.21 ± 2.14	23.89 ± 1.87	2.01 ± 0.83	3.03 ± 0.76	N/A	N/A
Huang (13)	16.67%	33.33%	9.44%	40.56%	42.22%	7.78%	35.56%	14.44%	N/A	N/A	N/A	N/A	N/A	N/A
Zhong et al. (37)	20.00%	30.00%	15.00%	35.00%	45.00%	5.00%	36.67%	13.33%	24.53 ± 1.72	19.37 ± 2.09	N/A	N/A	21.10 ± 2.54	29.93 ± 3.48
Zhuang (38)	N/A	N/A	N/A	N/A	N/A	N/A	N/A	N/A	N/A	N/A	N/A	N/A	14.75 ± 3.12	16.69 ± 3.51
Hu and Wen (39)	27.88%	22.12%	25.00%	25.00%	45.19%	4.81%	39.42%	10.58%	N/A	N/A	N/A	N/A	N/A	N/A
Xue (14)	18.00%	32.00%	4.00%	46.00%	48.00%	2.00%	39.00%	11.00%	25.08 ± 5.52	18.84 ± 6.62	2.24 ± 1.02	4.06 ± 1.05	N/A	N/A
Liu et al. (40)	36.00%	14.00%	21.00%	29.00%	46.00%	4.00%	37.00%	13.00%	N/A	N/A	N/A	N/A	N/A	N/A
Wei et al. (41)	32.50%	17.50%	12.50%	37.50%	48.75%	1.25%	40.00%	10.00%	N/A	N/A	1.59 ± 1.01	3.97 ± 1.35	N/A	N/A
Huang and Jing (32)	38.37%	11.63%	23.26%	26.74%	48.84%	1.16%	40.70%	9.30%	N/A	N/A	N/A	N/A	N/A	N/A
Qu (42)	30.00%	26.36%	11.82%	31.82%	54.55%	1.82%	36.36%	7.27%	N/A	N/A	N/A	N/A	N/A	N/A
Chen et al. (43)	5.00%	45.00%	0.00%	50.00%	48.75%	1.25%	23.75%	26.25%	21.50 ± 4.50	14.50 ± 3.20	N/A	N/A	N/A	N/A
He et al. (44)	16.67%	33.33%	5.00%	45.00%	46.67%	3.33%	41.67%	8.33%	N/A	N/A	N/A	N/A	N/A	N/A
Song and Luo (45)	30.00%	20.00%	23.75%	26.25%	48.75%	1.25%	37.50%	12.50%	N/A	N/A	N/A	N/A	N/A	N/A
Zhang et al. (46)	11.11%	39.68%	6.35%	42.86%	49.21%	1.59%	42.86%	6.35%	21.94 ± 2.55	20.84 ± 2.49	1.93 ± 1.06	2.79 ± 1.13	N/A	N/A
Zhou et al. (15)	14.63%	34.15%	8.54%	42.68%	45.12%	3.66%	40.24%	10.98%	N/A	N/A	N/A	N/A	N/A	N/A
Ma and Zhang (16)	28.79%	21.21%	15.91%	34.09%	46.97%	3.03%	38.64%	11.36%	N/A	N/A	N/A	N/A	N/A	N/A
Yao et al. (30)	20.83%	29.17%	15.83%	34.17%	47.50%	2.50%	40.83%	9.17%	N/A	N/A	N/A	N/A	N/A	N/A
Zhao (17)	13.51%	43.92%	3.38%	39.19%	47.30%	10.14%	25.68%	16.89%	N/A	N/A	N/A	N/A	N/A	N/A
Lai (47)	31.67%	20.56%	20.00%	27.78%	47.22%	5.00%	31.11%	16.67%	N/A	N/A	N/A	N/A	N/A	N/A

### 3.3 Risk of bias assessment

**Figure 2:**
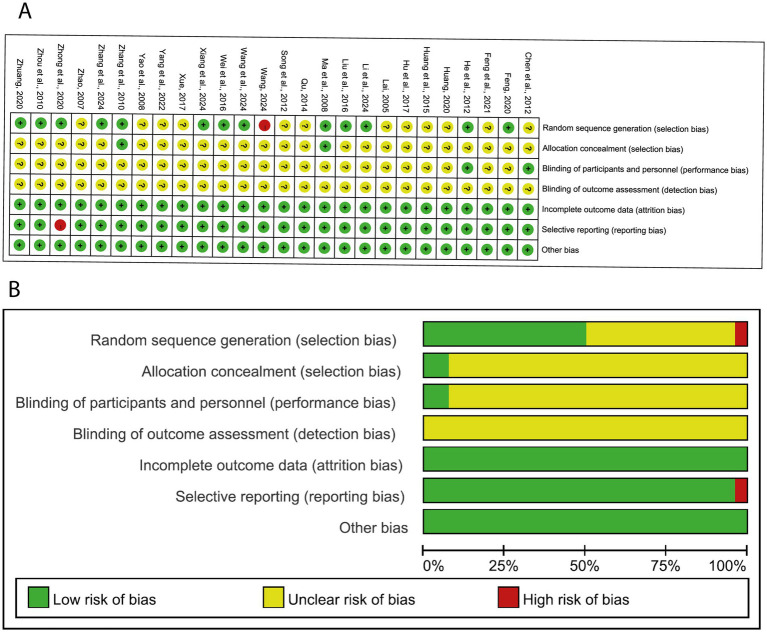
**(A,B)** shows the risk of bias assessment.

Figure 2 shows the risk of bias assessment. In terms of random sequence generation, 13 studies provided a sufficient randomization process to generate random sequences with a low risk of bias, and the remaining 13 studies supplied non-specific details of randomization and thus were assessed as exhibiting unclear risk. Two studies used an envelope method to perform the allocation concealment, while the other studies did not report the method. Only two studies described the implementation of single blinding of subjects, which was rated as low risk. None of the other included studies explicitly mentioned the use of the blind method, resulting in an unclear associated risk of bias. All studies included in the analysis published complete data regarding the outcomes, leading us to rate the risk of bias as low. For other biases, all of the studies were assessed as low risk.

### 3.4 Effective rate

**Figure 3:**
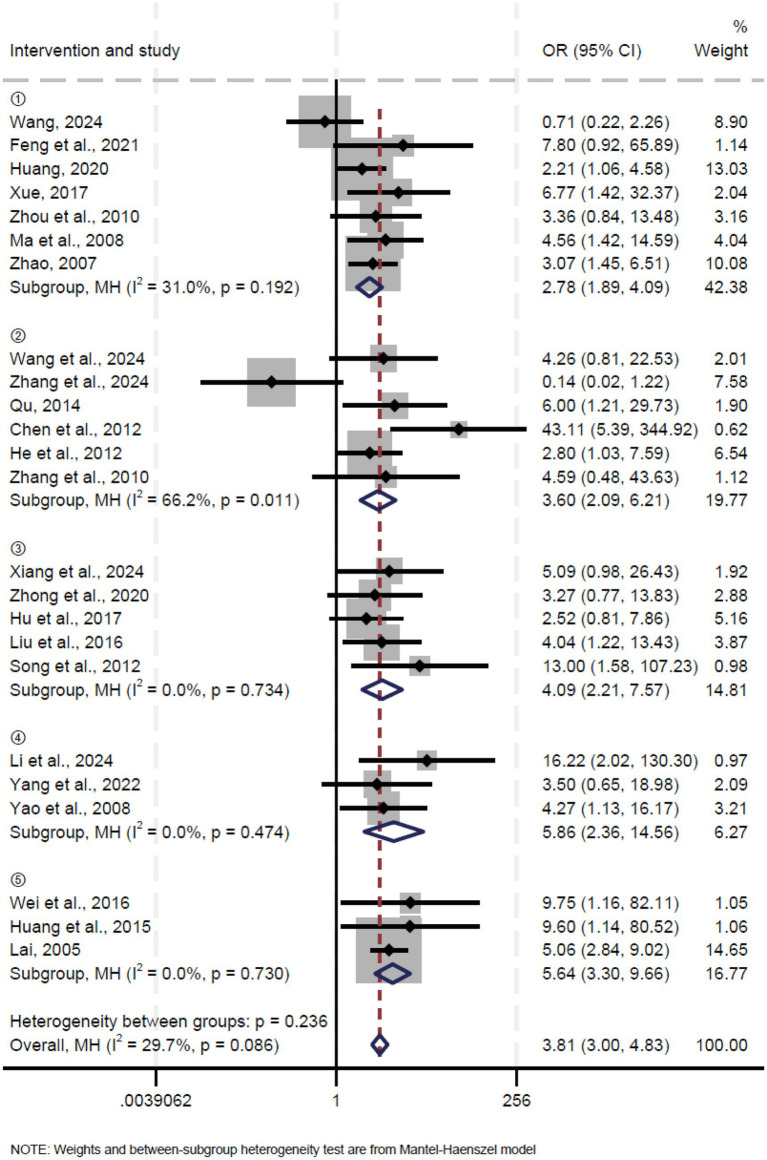
Forest plot of the cure rate comparing TCM with control interventions in patients with lumbar disc herniation.

A total of 24 studies reported the effectiveness rate. Pooled results showed that oblique pulling manipulation significantly improved the effective rate in patients with LDH compared to the control group (OR = 2.78, 95% CI: 1.89 ~ 4.10, *p* < 0.00001) with low heterogeneity (*I*^2^ = 31.0%, *p* = 0.192) (Figure 3). Similarly, the meta-analysis of oblique pulling manipulation combined with the massage group versus the control group showed a significantly higher effective rate (OR = 3.60, 95% CI: 2.09 ~ 6.21, *p* < 0.00001), and high heterogeneity was observed (*I*^2^ = 66.2%, *p* = 0.011) (Figure 3). Moreover, oblique pulling manipulation combined with acupuncture significantly improved the effective rate compared to the control group (OR = 4.09, 95% CI: 2.21 ~ 7.57, *p* < 0.00001) with low heterogeneity (*I*^2^ = 0.0%, *p* = 0.734) (Figure 3). In addition, both oblique pulling manipulation combined with Chinese herbal medicine (OR = 5.86, 95% CI: 2.36 ~ 14.56, *p* < 0.00001) and oblique pulling manipulation combined with injection therapy (OR = 5.64, 95% CI: 3.30 ~ 9.66, *p* < 0.00001) exhibited a higher effective rate compared to the control group with low heterogeneity (Figure 3). The results of the sensitivity analysis showed that excluding each study individually had no significant effect on the combined effect value, suggesting that the results of this meta-analysis were stable and reliable. The funnel plot analysis and Egger’s test suggested that there was no publication bias.

Potential sources of heterogeneity were explored by subgroup analysis based on sample size in the analysis of oblique pulling manipulation combined with the massage group versus the control group, and the results showed that the effective rate in patients with LDH in the large sample sizes (*n* ≥ 100) group was notably higher than the control group (OR = 3.47, 95% CI: 1.49 ~ 8.07) with high heterogeneity (*I*^2^ = 66.1%, *p* = 0.011) (Supplementary Figure S1), suggesting that sample size may not be a source of heterogeneity.

### 3.5 Cure rate

**Figure 4:**
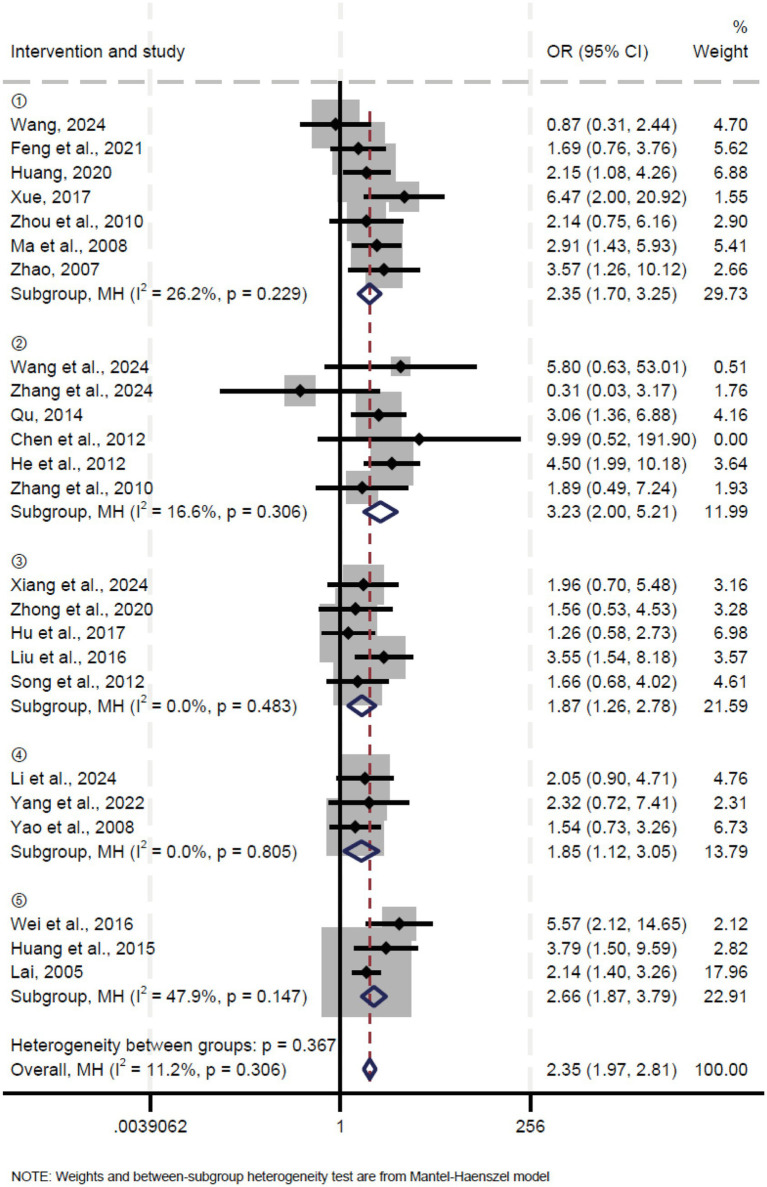
Forest plot of the effective rate comparing TCM with control interventions in patients with lumbar disc herniation.

A total of 24 studies reported the cure rate. Pooled results showed that oblique pulling manipulation significantly improved the cure rate in patients with LDH compared to the control group (OR = 2.35, 95% CI: 1.70 ~ 3.25, *p* < 0.0001) with low heterogeneity (*I*^2^ = 26.2%, *p* = 0.229) (Figure 4). Similarly, the meta-analysis of oblique pulling manipulation combined with the massage group versus the control group showed a significantly higher cure rate (OR = 3.23, 95% CI: 2.00 ~ 5.21, *p* < 0.0001), and low heterogeneity was observed (*I*^2^ = 16.6%, *p* = 0.306) (Figure 4). Moreover, oblique pulling manipulation combined with acupuncture significantly improved the cure rate compared to the control group (OR = 1.87, 95% CI: 1.26 ~ 2.79, *p* = 0.002) with low heterogeneity (*I*^2^ = 0.0%, *p* = 0.483) (Figure 4). In addition, both oblique pulling manipulation combined with Chinese herbal medicine (OR = 1.85, 95% CI: 1.12 ~ 3.05, *p* = 0.016) and oblique pulling manipulation combined with injection therapy (OR = 2.66, 95% CI: 1.87 ~ 3.79, *p* < 0.0001) exhibited a higher cure rate compared to the control group with low heterogeneity (Figure 4). The results of the sensitivity analysis showed that excluding each study individually had no significant effect on the combined effect value, suggesting that the results of this meta-analysis were stable and reliable. The funnel plot analysis and Egger’s test suggested that there was no publication bias.

### 3.6 Japanese Orthopedic Association (JOA) score

**Figure 5:**
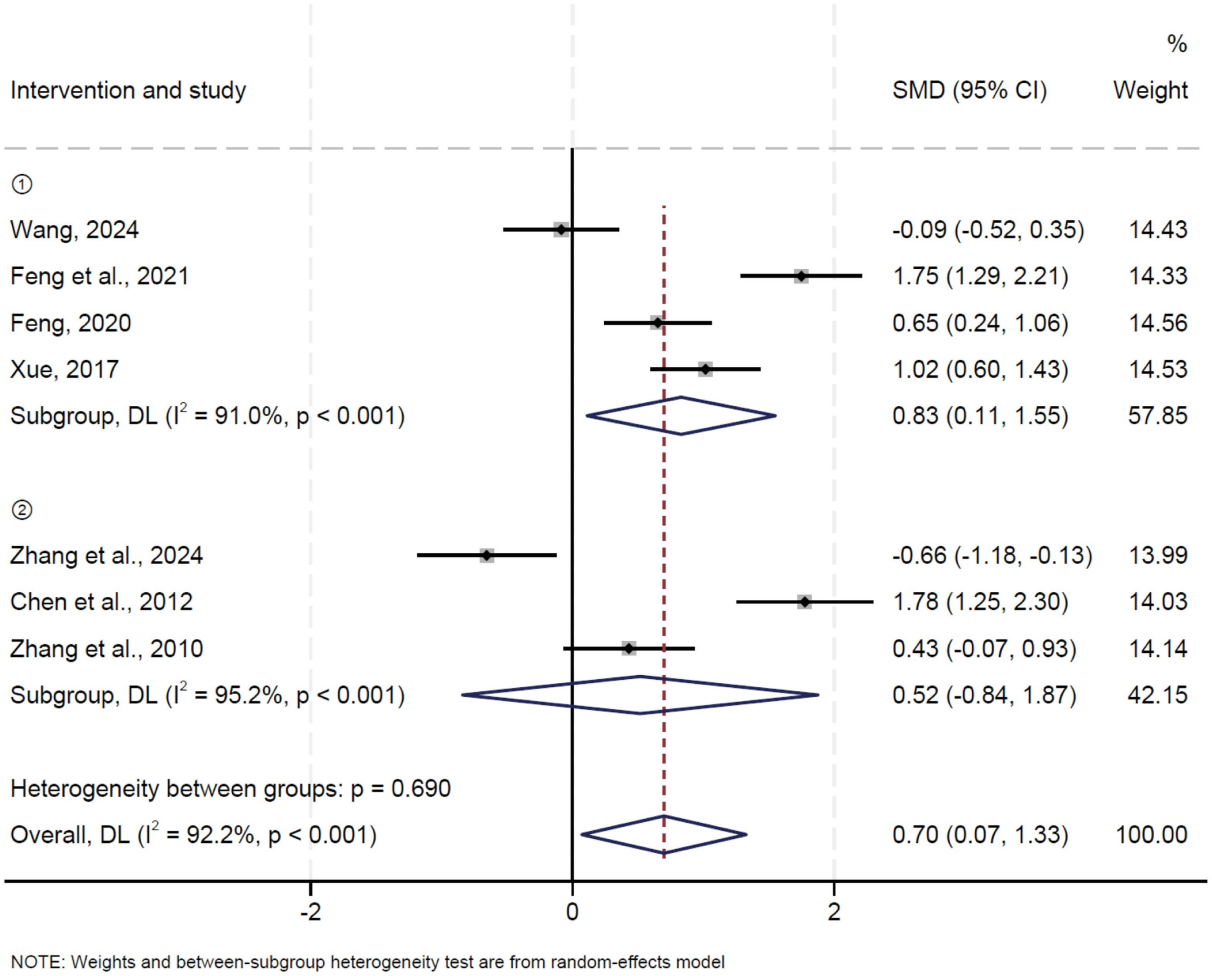
Forest plot of the JOA score comparing TCM with control interventions in patients with lumbar disc herniation.

A total of seven studies reported the Japanese Orthopedic Association (JOA) score. Pooled results showed that oblique pulling manipulation significantly improved the JOA score in patients with LDH compared to the control group (OR = 0.83, 95% CI: 0.11 ~ 1.55, *p* = 0.024) with high heterogeneity (*I*^2^ = 91.0%, *p* = 0.000) (Figure 5). No significant difference in JOA score was observed between the oblique pulling manipulation combined with massage group and the control group (OR = 0.52, 95% CI: −0.84 ~ 1.87, *p* = 0.455) (Figure 5). The results of the sensitivity analysis revealed that excluding each study individually had no significant effect on the combined effect value, suggesting that the results of this meta-analysis were stable and reliable. The funnel plot analysis and Egger’s test suggested that there was no publication bias.

Potential sources of heterogeneity were explored by subgroup analysis based on sample size in the analysis of oblique pulling manipulation versus the control group, and the results showed that the JOA score in patients with LDH in the large sample sizes (*n* ≥ 100) group was notably higher than the control group (OR = 2.38, 95% CI: 0.66 ~ 2.09) with high heterogeneity (*I*^2^ = 81.2%, *p* = 0.021) (Supplementary Figure S2), suggesting sample size may not be a source of heterogeneity.

### 3.7 Visual Analogue Score (VAS)

**Figure 6:**
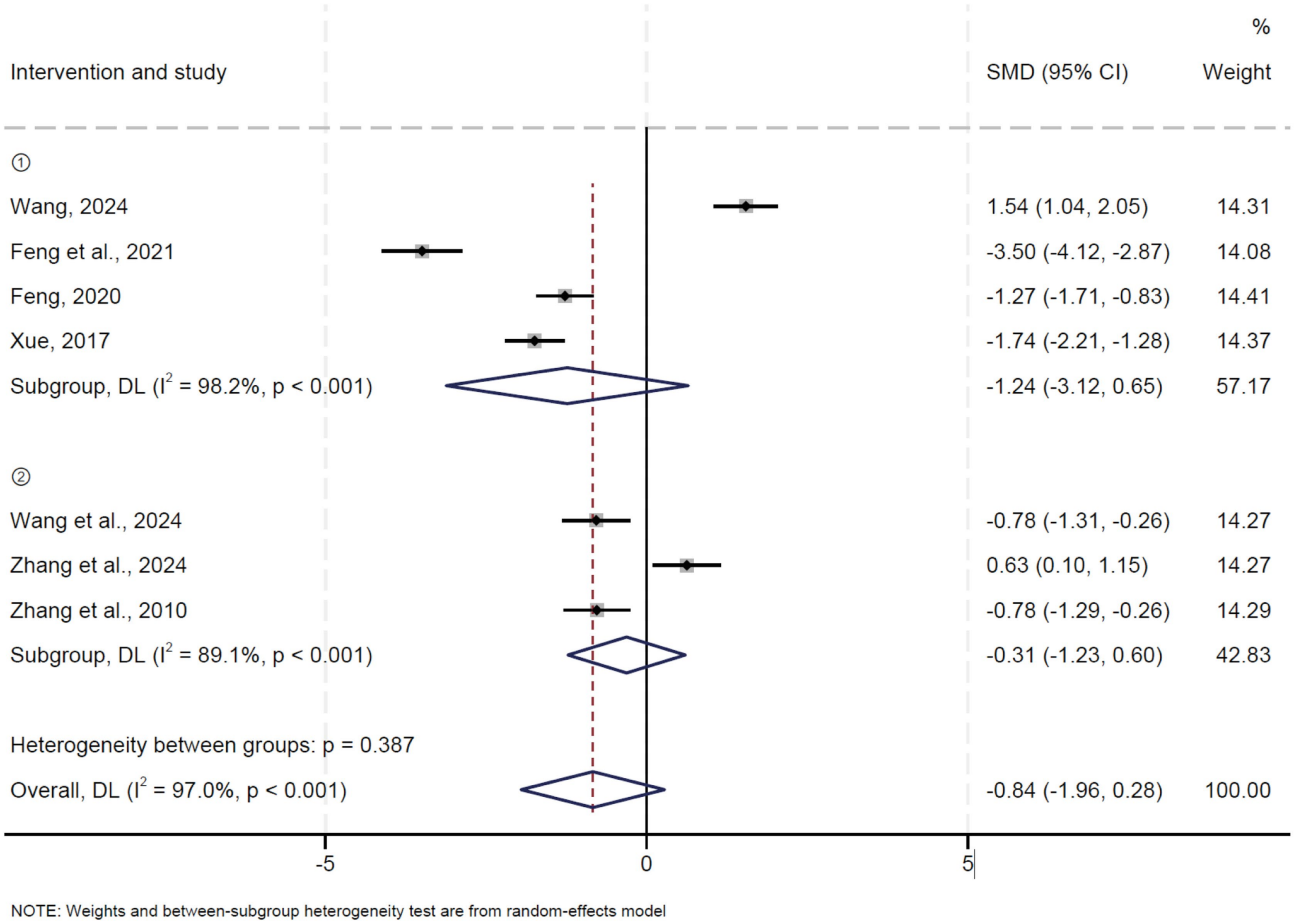
Forest plot of the VAS comparing TCM with control interventions in patients with lumbar disc herniation.

A total of seven studies reported the Visual Analogue Score (VAS). Pooled results showed that both oblique pulling manipulation (OR = −1.24, 95% CI: −3.12 ~ 0.65, *p* = 0.198) and oblique pulling manipulation combined with massage (OR = −0.31, 95% CI: −1.23 ~ 0.60, *p* = 0.502) had no significant effect on VSA in patients with LDH compared to the control group (Figure 6). The results of the sensitivity analysis showed that excluding each study individually had no significant effect on the combined effect value, suggesting that the results of this meta-analysis were stable and reliable. The funnel plot analysis and Egger’s test suggested that there was no publication bias.

### 3.8 Oswestry Disability Index (ODI)

**Figure 7:**
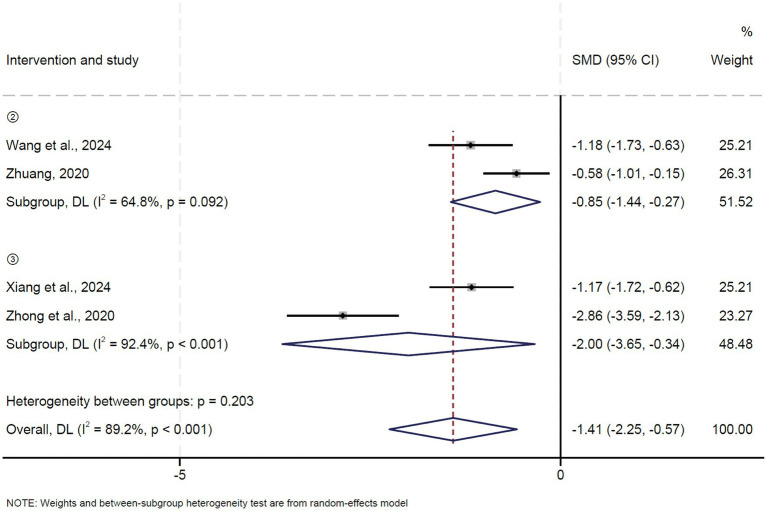
Forest plot of the ODI comparing TCM with control interventions in patients with lumbar disc herniation.

A total of four studies reported the Oswestry Disability Index (ODI). Pooled results showed that oblique pulling manipulation combined with massage significantly decreased the ODI compared to the control group (OR = -0.85, 95% CI: −1.44 ~ −0.27, *p* = 0.004) with high heterogeneity (*I*^2^ = 64.8%, *p* = 0.092) (Figure 7). Moreover, oblique pulling manipulation combined with acupuncture also significantly reduced the ODI compared to the control group (OR = −2.00, 95% CI: −3.65 ~ −0.34, *p* = 0.018) with high heterogeneity (*I*^2^ = 92.4%, *p* < 0.0001) (Figure 7). The results of the sensitivity analysis showed that excluding each study individually had no significant effect on the combined effect value, suggesting that the results of this meta-analysis were stable and reliable. The funnel plot analysis and Egger’s test suggested that there was no publication bias.

## 4 Discussion

The current research study aimed to evaluate the clinical efficacy of oblique pulling manipulation and its combination with massage, acupuncture, Chinese herbal medicine, and injection therapy in lumbar disc herniation (LDH). The meta-analysis results revealed that oblique pulling manipulation and its combination with massage, acupuncture, Chinese herbal medicine, and injection therapy in LDH significantly improved the effective rate and cure rate in patients with LDH. Oblique pulling manipulation significantly improved the Japanese Orthopedic Association (JOA) score, and oblique pulling manipulation combined with massage or acupuncture decreased the Oswestry Disability Index (ODI). Oblique pulling combined with injection therapy yielded the highest cure rate (38.4% vs. 23.3%), but standardized protocols and long-term safety data are urgently needed. In addition, no RCTs reported adverse events.

LDH is the most common spinal degenerative disorder, characterized by persistent or recurrent radicular pain and positive nerve-root tension signs, significantly impacting their daily lives and work (19). The main clinical treatments for LDH are conservative treatment and surgical treatment. In traditional Chinese medicine (TCM), manual therapy is one of the most commonly used complementary therapies for LDH, and oblique pulling manipulation is a typical manual therapy to achieve appreciable pain relief and function improvement (20). Oblique pulling manipulation alleviates the symptoms of LDH through multiple possible mechanisms: applying counterpressure to the ipsilateral hip and shoulder reduces intradiscal pressure, enlarges the nerve-root canal, and releases adhesions in the facet joints; shifting between the herniated disc and adjacent nerve roots, thereby reducing neural compression and alleviating pain; and relaxing paraspinal soft tissues and improving lumbar flexibility and relief of low back pain (9).

The current findings are consistent with the previous results on LDH, in which oblique pulling manipulation improved the effective rate and cure rate and increased the JOA score after treatment (11, 12). Studies have demonstrated that combining oblique pulling manipulation with massage significantly alleviates clinical symptoms in LDH patients; the benefits might arise from enhanced local circulation, analgesic action, unblocking of meridians and collaterals, and restoration of paraspinal muscle flexibility and coordination (21, 22). However, an onset of cauda equina syndrome following massage has been reported (23, 24), while a recent study demonstrated that chiropractic spinal manipulation is not a risk factor for cauda equina syndrome; patients who developed cauda equina syndrome following chiropractic spinal manipulation may have had symptoms and/or preexisting disc herniation before treatment (25). The combination of oblique pulling manipulation and acupuncture not only enhanced lumbar spine function but, through the thermal effect of warm needling, but also improved local microcirculation and accelerated the absorption of inflammatory mediators (26). Acupuncture may reduce inflammation via IL-6 suppression (27, 28). When combined with oblique pulling manipulation, Chinese herbal medicine synergistically warms and unblocks the meridians, dispels wind, cold, and dampness to relieve pain, and exerts anti-inflammatory and analgesic effects by inhibiting pro-inflammatory mediators, thereby reducing local swelling and pain (29–31). Moreover, combining oblique pulling manipulation with epidural or paravertebral corticosteroid injections produced significant clinical benefits (32). Dexamethasone, in particular, demonstrated potent anti-inflammatory and anti-fibrotic effects, reduced disc-induced exudation, scavenged free radicals, and prevented perineural adhesion (33–35).

There are several limitations to the current study. Some of the included studies did not report key outcome measures such as the JOA score, the ODI, and the VAS, hindering a comparative analysis of treatment effects on these metrics. Another limitation was the relatively small overall sample size, as the 26 included studies collectively enrolled 2,766 patients. Furthermore, none of the included trials reported any adverse reactions. The safety profile of oblique pulling manipulation in LDH treatment thus remains undetermined.

## 5 Conclusion

Oblique pulling manipulation and its combination with massage, acupuncture, Chinese herbal medicine, and injection therapy in LDH significantly improved the effective rate and cure rate in patients with LDH. The rare cases of cauda equina syndrome reported after oblique pulling manipulation underscore the need for meticulous procedural technique and vigilant neurological monitoring. Future research should establish standardized treatment protocols and extend follow-up durations to ensure broader applicability and better patient outcomes.

## Data Availability

The original contributions presented in the study are included in the article/Supplementary material, further inquiries can be directed to the corresponding author.
