# Supplementary material for: Efficacy of oblique pulling manipulation combined with adjunct therapies (massage/acupuncture/herbal medicine/injection) for lumbar disc herniation: a systematic review and meta-analysis of pain and functional outcome
Source: Front Neurol. 2026 Jan 19;16:1700862. doi: 10.3389/fneur.2025.1700862 (PMC12862601; doi:10.3389/fneur.2025.1700862)

Supplementary Material

Figure S1. Subgroup analysis of the effective rate in patients with lumbar disc herniation between the oblique pulling manipulation combined with massage group and the control group.


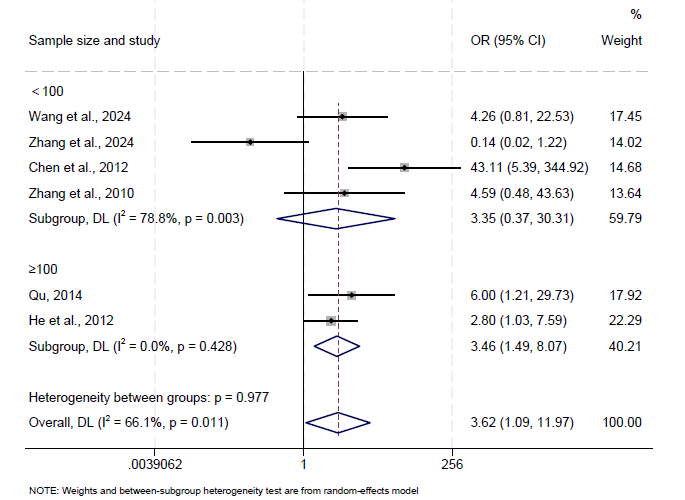


Figure S2. Subgroup analysis of the JOA score in patients with lumbar disc herniation between the oblique pulling manipulation group and the control group.


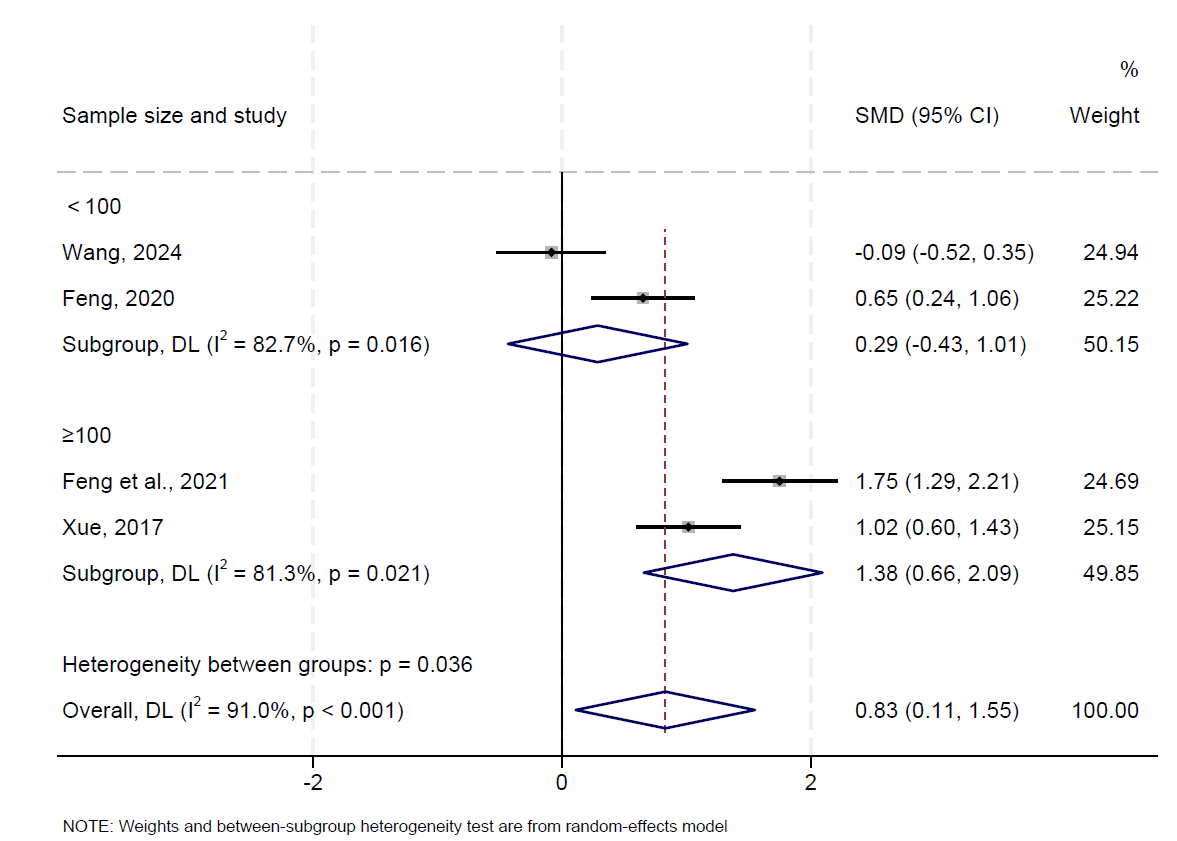

Supplement: Supplementary file 1 [file Table_1.docx]
